# Characteristics of CD44 alternative splice pattern in the course of human colorectal adenocarcinoma progression

**DOI:** 10.1186/1476-4598-11-83

**Published:** 2012-11-14

**Authors:** Balázs Bánky, Lívia Rásó-Barnett, Tamás Barbai, József Tímár, Péter Becságh, Erzsébet Rásó

**Affiliations:** 1 2nd Institute of Pathology, Semmelweis University, Üllői út 93., 1091, Budapest, Hungary; 2 St Borbála Hospital, Department of Surgery and Vascular Surgery, Dózsa Gy. út 77., 2800, Tatabánya, Hungary; 3 Guy’s and St. Thomas’ Hospital, Westminster Bridge Road, London, SE1 7EH, United Kingdom; 4 Tumour Progression Research Group, Hungarian Academy of Sciences, Budapest, Hungary

**Keywords:** CD44, v3, v6, Alternative, Splicing, Metastasis, Colorectal, Cancer, Microenvironment

## Abstract

**Background:**

CD44 is considered as ‘a’ metastasis associated gene, despite the fact that it is an umbrella term for a group of molecules produced from a single gene by alternative splicing. However, little consideration is given to the above in the literature of colorectal carcinomas as well as other tumour types, leading to confusion and contradictory results about its possible role in tumour progression.

**Methods:**

We compared the CD44 alternative splice pattern (ASP) of three genetically different human colorectal cancer cell lines (HT25, HT29, HCT116) using a series of PCR reactions and next- generation sequencing method, as well as identified a colorectal adenocarcinoma specific CD44 ASP. This ASP was further investigated in terms of its qualitative and quantitative stability in our experimental iso- and xenograft mouse models for colorectal cancer progression. A complex preclinical experimental set-up was established to separately test the different steps of tumour progression and the role of tumour microenvironment, respectively, focusing on the role of ‘CD44’ in this process.

**Results:**

We managed to present a colorectal cancer-specific CD44 ASP, which remained unchanged from cell lines throughout primary tumour formation and metastatic progression. Furthermore, we report a unique roster of all expressed CD44 variant isoforms characteristic to colorectal cancer. Finally, on quantitative assessment of the variable exons v3 and v6, higher co-expression levels were found to be characteristic to metastatically potent tumour cells.

**Conclusion:**

Particular CD44 variant isoforms seem to act as “metastasis genes” via tumour microenvironment-driven shifts in v3 and v6 expressions. However, this function may just affect a minority of tumour subclones. This fact and the huge potential number of different CD44 splice variants that can contain v3 and v6 domains can explain incoherence of clinical studies regarding functional asessment of CD44 variants, as well as diminish the chances of using CD44 variants for predictive purpose.

## Background

In developed countries, colorectal cancer (CRC), or rather its progression to metastatic disease, accounts for 25% of tumour deaths [1]. Characterising the metastases associated processes is therefore of crucial importance for identifying ways of earlier and more sensitive diagnosis, more defined prognosis, and possibly in the selection of patients for targeted therapies.

A variety of genes have been described and extensively investigated in the literature as key candidates in the tumorigenesis and progression of colorectal carcinoma, including APC, p53, K-ras, BRAF, DCC, MSH, EGFR, SFK, TGFR2, SMAD4, etc. [2-5].

Each individual step of the metastatic cascade is the result of complex molecular interactions, regulated by several already identified and/or unidentified genes [6]. One of the candidates of key importance has been CD44. In fact, it is one of the most investigated molecules in the metastatic process of several malignancies [7-13], among them that of colorectal cancer [14-16].

CD44 was first described to be a lymphocyte homing receptor [17]. The role of CD44 was later proven in fetal arteriogenesis [18], as well.

Furthermore, expression and function of various isoforms of CD44 were detected in chronic inflammatory diseases (rheumatoid arthritis, ulcerative colitis, resolution of lung inflammations, etc.) [19-21].

A number of studies demonstrated the expression of CD44s (standard version) on most tissue types of epithelial origin (stomach mucous membrane, small intestinal mucosa, prostate, ductal epithelium of breast, skin, hair follicules, transitional cell membrane of utgenital tract) [22-26]. Regarding colonic mucosa, immunohistochemical examinations demonstrated that CD44s (identified with antibody against the standard region of the molecule) is located at the level of basal crypts, similar to the “intestinal stem cells” of the small intestine sitting between Paneth-cells [27-29]. This means that proliferative cells of colonic mucosa, as well as cells in the basal segment of differentiation zone are able to provide CD44s expression, while differentiated mucosal cells don’t show membrane positivity on immunostaining any more [30]. This fact gains special importance when considering the change of ‘CD44’ expression characteristics from the normal, the dysplastic and the malignant colorectal tissues [31].

Additionally, and probably not independently form the above mentioned findings, recent studies demonstrated ‘CD44’ as a potential tumour stem cell marker (in colorectal, gastric and pancreatic adenocarcinoma, as well as breast cancer and other cancers) [32-39].

With the presence of CD44 protein in so many tissues, consideration has to be given to its physiological and pathological functions.

‘CD44’ has a variety of biological functions such as cell-cell, cell-ECM interactions, tumour cell migration [40,41] or even chemoresistance [16,41-50]. This multitude of representations and functions is less surprising when considering that through its 9 variable exons (v2-v10), hundreds of different isoforms can be transcribed and translated from the single CD44 gene resulting in a mixture of glycoproteins with potentially different functions [51-53]. Talking about ’CD44’ and studying its ’over expression’ therefore is rather debatable and not as straight-forward as it was previously thought. A basic understanding and consideration of alternative splicing should be warranted when dealing with this versatile gene. For the same reason, when using probes (i.e. primers or antibodies) against the standard region of CD44 (CD44s), one will detect a mixture of isoforms as they all share the same standard region [54,55].

Determining the role of ’CD44’ is further complicated by the difficulties to identify all the isoforms potentially characterizing certain cell or tissue types. The trend is to focus on the expression of a single variable exon, which, of course, will only show the summation of isoforms containing the exon in question. However, these isoforms might only share a single variable exon, therefore they can be of different functions. In addition, several other CD44 variant isoforms can be present within the same cell, adding further functions to ’CD44’ [56-59]. Furthermore, different cells of a tumour can express various, possibly different sets of CD44 isoforms. As most of our tests examine a group of cells from the tissue at the same time, the results are summated, failing to represent the cell-to-cell differences in details [6]. In colorectal carcinoma the most studied variant exons / protein domains are v3 and v6.

CD44v3 is the heparane-sulphate proteoglycane domain of CD44, however, the v3-containing isoforms of CD44 have hyaluronate binding potential and may contain a chondroitin-sulphate proteoglycan domain as well. Consequently, CD44v3 can bind different heparin-binding growth factors (GF), such as HBEGF, VEGF, HGF, βFGF, KGF and amphiregulin [60,61].

CD44v6 functions as receptor of HGF/SF cooperating with c-met [61,62], regulating EMT and taking part in the positive feedback loop of K-ras activation driving both Ras signal transduction pathway and CD44-splicing machinery [3,63,64]. Additionally, this is the domain most frequently found to be associated with metastatic phenotype in the literature [65].

Unsurprisingly, the studies of detecting ‘v3’ and ‘v6’ under different experimental and clinical settings do not lead to coherent results, and can even be contradictory. This also can be observed in the literature regarding colorectal cancer [14,66-71].

Recently, characteristic CD44 isoform patterns of different normal tissue types have been described in the literature [24-26,72,73] and our earlier studies described CD44 isoform patterns characteristic of different tumour types, respectively (data publication in progress, Rásó-Barnett et al.). Most of the literature agrees that ‘CD44’ plays an important role during tumour progression based on ‘overexpression’ of specific variant regions containing isoforms at mRNA and/or protein level. However, no data is available concerning the pattern of CD44 isoforms and its modulation during tumour progression. Our previous study on CD44 alternative splice patterns showed no qualitative change of the melanoma specific ‘CD44-fingerprint’, while the expression level of all isoforms uniformly increased during the metastatic process (data publication in progress, Rásó-Barnett et al.).

The aim of our research was to find answers to the following two questions: (1) Is there a colorectal carcinoma specific CD44 isoform expression combination like to one identified in human melanoma? If there is such pattern, is it qualitatively / quantitatively stable during tumour progression? (2) What is the background of the incoherent quantitative results on the expression of the most investigated v3 and v6 variable exons / protein domains?

To answer the first question, we designed primer pairs covering the entire variable region (primers A-B, see Methods) for our quantitative and qualitative PCR reactions. The expressed, i.e. transcribed CD44 isoforms were identified using direct sequencing and next-generation sequencing.

Due to the large number of potential isoforms, we used a series a PCR reactions to create the CD44 fingerprint, a simple, easy to handle representation of the expressed CD44 isoforms. We used this fingerprint to track the changes of CD44 expression during tumour progression [41,55,74-76].

To answer our second question, we designed a complex experimental animal model to examine the functional role CD44v3 and CD44v6 containing isoforms play in metastasis formation. The main question was whether or not there is a change in the expression of these variable exons that could be associated to the metastatic process of human colorectal carcinoma. We implanted the same colorectal cancer cell population orthotopically to form real metastases in liver, as well as heterotopically. We performed two types of heterotopic implantation: the first involved subcutaneous implantation into permissive and non-permissive hosts, the second was direct implantation into the spleen so that the implanted cells could directly colonize the liver. The latter method created a new type of secondary tumour, which albeit is in the same localization as the liver metastases of the true metastatis model, has not been through the metastatic cascade. This experimental setup allowed to functionally differenciate between phases of metastatic process and host related changes, regarding the expression activity of the examined variable CD44 exons.

## Results

### Colorectal adenocarcinoma (CRC) specific CD44 alternative splice pattern (ASP)

**Figure 1:**
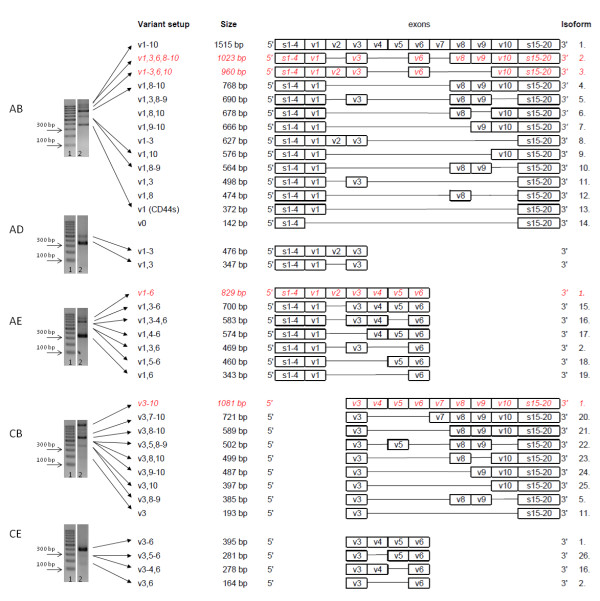
**Expressed CD44 isoforms in colorectal cancer.** Agarose gel electrophoretogram of PCRs with primer combination **A**-**B**, **A**-**D**, **A**-**E**, **C**-**B**, **C**-**E** on human CRC cell line HT29. (1. 100bp DNA ladder; 2. CD44 expression pattern of HT29 suspension). Primer design shown on Figure 2A. Expressed CD44 isoforms in colorectal cancer were detected. PCR with **A**-**B** primer pair can theoretically provide all expresed isoforms, but for the reason of sensitivity limitations of PCR technique only five main bands were detected. PCRs with the other four primer pairs could reveal expressed (quantitatively inferior) isoforms containing v3 and v6 exon products.Some of the bands could be identified as distinct isoform products (proven by direct sequencing), others covered more, similar length isoforms identified by allele-specific next-generation sequencing or estimated by length calculation of the products. Estimated/calculated isoforms are shown in Italic, red letters. Twenty-six CD44 isoforms could be differentiated this way, some of them are just truncated, so that may in fact cover several isoforms sharing the same common variant exons.

**Figure 2:**
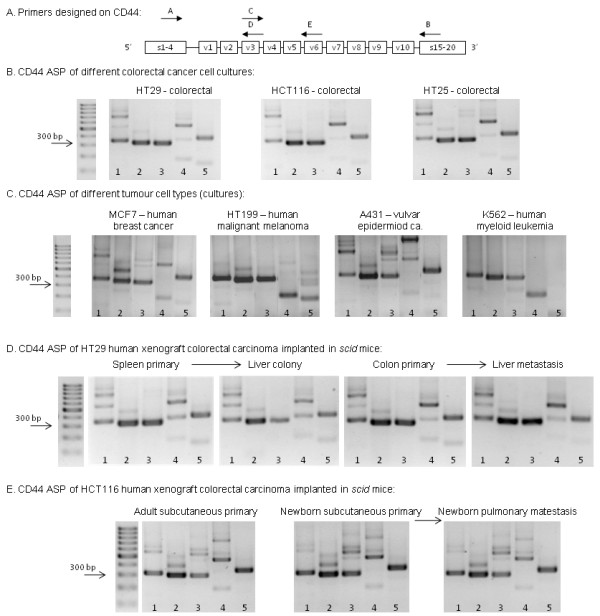
**Agarose gel electrophoretograms demonstrating CD44 isoform expression pattern of human CRC (colorectal specific "CD44 fingerprint"). A**. Primers designed on CD44 (Primer assembly: 1. **A**-**B**; 2. **A**-**D**; 3. **A**-**E**; 4. **C**-**B**; 5. **C**-**E**). **B**. ’CD44 fingerprint’ of colorectal cancer suspensions (HT29, HCT116 and HT25) in vitro A highly similar pattern of the expressed CD44 isoforms can be recognised. **C**. CD44 fingerprint of different human malignant cell line types: MCF7 human breast cancer cell line, HT199 human malignant melanoma cell line, A431 human vulvar carcinoma cell line and K562 human myelogenois leukemia cell line, respectively. **D**. CD44 fingerprint of HT29 colorectal cancer xenografts in orthotopic implantation model and liver colonization model. Both primary colon tumours and their liver metastases, as well as spleen primary tumours (heterotopic implantation of colon tumour suspension) and the developed liver secondaries swow the same CD44 isoform expression pattern. **E**. CD44 fingerprint of HCT116 xenografts in spontaneous pulmonary metastasis model of subcutaneous tumour implantation: subcutaneous primary tumours in adult and in newborn, as well as pulmonary metastases in newborn scid mice expressed the same, colorectal carcinoma specific CD44 fingerprint on serial PCR and agarose gel ELFO. (Adult mice after subcuticular tumour implantation never developed pulmonary metastases).

Human colorectal cell lines (HT25, HT29, HCT116) could be characterized by the dominance of five bands on agarose gel electrophoretograms (ELFO) when examining the variant region of CD44 with a primer pair testing and overlapping the entire variant region (Primer A – Primer B) Figure 1, Figure 2B. This PCR reaction would theoreticaly amplify each transcribed CD44 variant isoform. In three of the five bands (372 bp, 564 bp, 768 bp) we identified a particular CD44 isoform by direct sequencing. Even these bands, as well as those bands which were not definitely identified by direct sequencing, potentially ‘hide’ more, different isoforms. None of the dominant PCR producs that were identified by direct sequencing represented v3 or v6 containing isoforms. Next-generation sequencing (NGS) performing allele specific high performance sequencing managed to identify further isoforms including some of those which contain v3/v6 exon products. This method managed to differentiate more isoforms hidden behind the same ELFO bands. Furthermore, eight more isoforms with very low expression activities were identified by this sensitive method. These isoforms do not appear on the qualitative picture of ELFO. (Surprisingly, NGS provided evidence to the fact that CD44 isoform without any variant exons (CD44v0) does exist, in spite of the widely accepted theory that v1 variant exon is constitutively expressed) Figure 1.

Additionally, we identified a number of *definitely* expressed CD44 isoforms by four further PCR sets performed with primer pairs directly designed onto exons v3 and v6 (Primer pairs A-D, A-E, C-B, C-E) Figure 1, Figure 2A. Primers designed for particular exons (e.g. v3 or v6) gave evidence of that exon being transcribed, as a minimal premise of amplification on PCR. Some of these isoforms did not appear among the products of the reaction ‘A-B’, either for some quantitative or particular PCR reaction technical reasons. Precise knowledge of exon lengths enabled us to calculate the expression of further isoforms according to product sizes in addition to the ones detected by direct sequencing of PCR products. These two methods provided a rich roster of definite and estimated CD44 isoforms which are represented by being isoforms of very similar size.

This way we identified CD44 isoforms expressed in CRC cell lines from a qualitative picture derived from five-primer-pair PCR series covering the entire length of the variable region of CD44 with overlapping sequences. By running the PCR products of the five different primer pair reactions on agarose gel always in the same order, a colorectal cancer specific alternative splice pattern (ASP) could be described for all three human colorectal cell lines (HT29, HCT116 and HT25) Figure 2B.

Colorectal specificity of this CD44-fingerprint is detailed and proven elsewhere (publication in progress, Rásó-Barnett et al.), here we just demonstrate a few samples of CD44 fingerprints of other tumour types, i.e. MCF7 (breast cancer), HT199 (melanoma), A431 (vulvar epidermoid carcinoma), and K562 (myelogenous leukemia) respectively.

After all, there were 26 different CD44 isoforms identified in the qualitative CD44 fingerprint of colorectal cancer, 24 of which were proven by sequencing and only two remained ‘estimated/calculated’ isoforms Figure 1. However further isoforms can also be present, only hidden behind ‘truncated’ CD44 isoforms sharing the same truncated part of the whole molecule (i.e. Isoform 14–26 on Figure 1).

### Characterisation and examination of the stability of the colorectal CD44 ‘fingerprint’

#### Comparison of tumours growing in vitro and in vivo

CD44 pattern of our samples from the xenotransplantation model has proven that the pattern is preserved in vivo the same way it was seen in vitro. ASP of CD44 was found to be highly similar of HT29 cells cultured in vitro as compared to orthotopic primary (colon) or heterotopic primary (spleen, subcutaneously in adult and newborn mice) Figure 2D,E. HT25 and HCT116 cell lines were found to behave the same conservative way, (data not shown).

#### Behaviour of CRC-specific CD44 alternative splice pattern (ASP) during the metastatic process of the tumours

To determine whether the expression pattern changes during the metastatic process in our xenograft system, we implanted HT29 cell suspension into spleen and orthotopically into large bowel, coecal wall. We then compared the CD44 pattern of the primary tumour and the liver metastasis/colony of the same animal in both models. The pattern was found to be unchanged Figure 2D. Similar, unchanged expression patterns were detected in the other two tumour types, HT25 and HCT116, respectively (data not shown).

In the case of HT29, the heterotopically (subcutaneous) implanted primary tumour showed the exact same pattern as the cell suspension in both the metastatic (newborn) and non-metastatic (adult) host. In metastatic cases (in each newborn animal) pulmonary metastases gave the same ASP, as well Figure 2E. The number of implanted animals in each arm of the model was three, the qualitative expression pttern was overally homogenous. (A total number of 40 animals were transplanted, 28 of them developed metastases, and we used 3–3 animals to check the CD44 fingerprint.)

### Quantitative change of certain exons (v3/v6) of the CD44 variable region during tumour progression of colorectal cancer in mice

#### Mouse C26 isograft system

**Figure 3:**
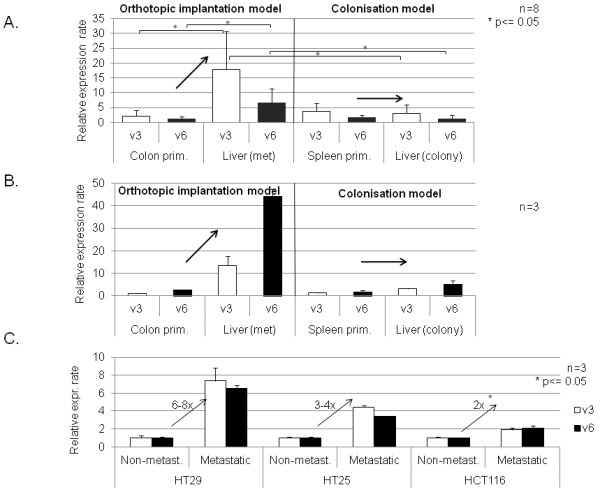
**CD44v3 and CD44v6 expression activity in mouse isograft CRC metastatic and liver colonisation system. A**. Semi-quantitative CD44v3 and v6 gene expression activity of primary and secondary *C26* colon carcinoma isograft tumours implanted orthotopically (colonic) and intrasplenic (liver colonisation model) into Balb/C mice.While in „proper” metastatic system (orthotopic implantation) liver metastases expressed CD44v3/v6 at significantly higher level than colon primaries, in colonisation model liver colonies showed similar expression profile to spleen primaries. **B**. CD44 v3 and v6 gene expression activity of primary and secondary *HT29* human colon carcinoma xenografts in orthotopic and intrasplenic implantation models to scid mice. Selection of CD44v3/v6 high subclone at primary site can be suspected, as liver metastases in orthotopic implantation model system showed higher expression activities than colon primaries. No „clone selection” effect was detected in liver colonisation model (after intrasplenic implantation). **C**. CD44v3 and v6 gene expression activity of non-metastatic (implanted into adult) and metastatic (implanted into newborn) primary human colon carcinoma xenograft tumours implanted subcutaneously (heterotopicaly) into *scid* mice. Expression rates are normalized on beta-actin expression rates and non-metastatic v3/v6 expression rates. All three examined human colorectal carcinoma cell types showed higher CD44v3 and CD44v6 expression activities in metastatic system (subcutaneously implanted colon tumours in newborn mice, which developed distant metastases in each animal cases) than in non-metastatic (the same colon carcinomas implanted into adult mice subcuticularly without any single case of distant metastasis formation).

CD44v3 and CD44v6 expressions of orthotopic (coonic wall) and heterotopic (splenic implantation) primary C26 tumours (four mice in each group), as well as spontaneous liver metastases and liver colonies (from splenic implantation) were compared using real-time PCR Figure 3A. Data indicate that while the expression level of both variable exons were more than one magnitude larger in the liver metastases compared to that of the primary tumour in the orthotopic implantation model (significant differences both on v3 and v6 expression), no significant alteration was detected between the spleen primary and the liver colony. Furthermore, it is remarkable that primary tumours of both systems showed similar expression levels.

#### Human colon cancer xenografts

All three implanted human colon cancer lines (HT29, HT25, HCT116) possessed similar metastatic potential in both systems, showing a 50–65 % metastatic rate.

Real time PCR measurement using CD44v3 and CD44v6 exon specific primers showed three different ways of behaviour in the three colorectal cell types. In representative samples we compared the relative CD44v3 and CD44v6 levels of the primary tumours and liver metastases or liver colonies in both systems, similarly to the isograft experiments Figure 3B.

In case of HT29, identically to C26, we detected significantly elevated expression levels in liver metastases after orthotopic implantation compared to the primary tumour of the colonic wall, while both variable exons were expressed at the same level in both the primary splenic tumour and the liver colony of the spleen-liver system. It is also interesting that primary tumours from both localisations showed similar expression levels.

**Figure 4:**
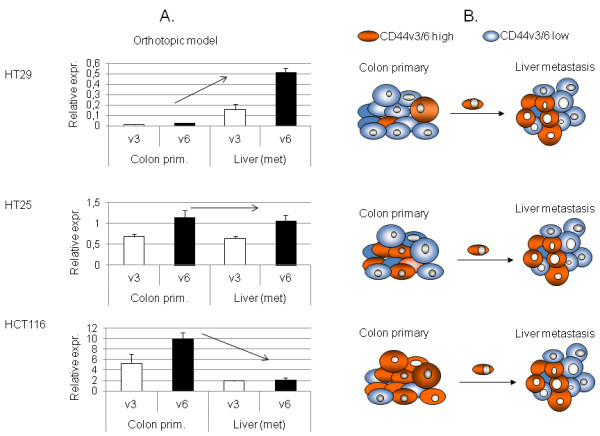
**CD44v3 and CD44v6 expression activity in human xenograft CRC metastatic and liver colonisation system. A**. CD44v3 and v6 gene expression activity of primary and metastatic human colon carcinoma xenograft tumours (HT29, HT25 and HCT116) implanted orthotopically (colonic wall)into scid mice. The three genetically different human colorectal carcinoma lines showed three different behaviours in terms of CD44v3 and v6 expression rate changes during metastatic process. The reason of it might be the baseline level of expression in prmary tumours: relative expression rates (CD44v3 and v6 expression normalized on β-actin expression rates) of the three tumour types showed 1–1 order of magnitude differences. As we measure summated expression rates (average) of each tumour cells of the particular tumour mass, differences in CD44 variant exon expression levels might be explined with differences of CD44v3/v6 high cell proportion of the tumour cell mass. **B**. Model of metastatic clone selection in each three cell lines, schematic explanation of the differences in a consequent model.

HT25 behaves opposite to Balb/C-C26 isograft system. Primary tumours and metastases both showed identical expression levels in the orthotopic system.In the case of HCT116, the primary tumours provided extremely high CD44v3/v6 expression levels. Liver metastases showed a magnitude lower expression level. Therefore, the third cell line represents a completely different, third pattern of behavior Figure 4A.

#### Spontaneous lung metastasis xenograft model from subcutaneous implantation

The same three, genetically different human colorectal cell suspensions (HT25, HT29, HCT116) were implanted into lumbar subcutaneous localization of three-three adult and newborn scid mice. No metastases were formed in the adult animals, while each newborn developed distant lung metastases. CD44v3 and CD44v6 expression levels of non-metastatic adult and metastatic newborn primary tumours were measured. The results from the three examined human xenografts are summarised on Figure 3C.

The difference in v3 and v6 variant expression level between the metastatic newborn and non-metastatic adult primary tumours is clearly demonstrated. The tendency is uniform across all three CRC tumours in this model, showing 2–3 fold higher v3 and v6 expression levels in the newborn than in adult primary tumours, however the difference with three animals in each arm could just reach the level of significance in the case of HCT116 colorectal tumour type.

## Discussion

Despite hundreds of published studies on CD44 within the last two decades, noconsensus opinion has been reached as of today apart from that it plays some role in tumour progression [69,76]. One of the possible explanations is that from microarrays to immunohistochemistry, the overwhelming majority of studies fail to take into consideration that ‘CD44’ is a collection of isoforms generated via alternative splicing of its nine variable exons. Probes, primers and antibodies against the standard region will recognise a pool of different isoforms as they all share the same standard region, and not the isoform itself, which is functionally active and involved in the metastatic process. A good example of this is colorectal adenocarcinoma, where the cells of origin, i.e. the glandular epithelium of the large bowel, express standard form of CD44 (CD44s), but not variant ones (CD44v), while highly dysplastic colorectal adenomas, primary and metastatic colorectal cancers express a large variety of different-length CD44 isoforms, both on mRNA and protein levels [14,31,44,77-79]. Some of these new CD44 isoforms acquired during malignant transformation contain v3 and v6 domain which add a completely different range of functions to the pre-existing CD44s functions, as CD44v6 is a co-receptor of the met protein, and CD44v3 is one of the most important presenters of heparin-binding growth factors, such as HGF, the main ligand of Met [60,62]. One must also consider that multiple variable regions might be present in a single isoform, adding further functional abilities. It is likely that the molecular interactions of CD44v3 are different from those of CD44v2-v10 variant despite both containing the v3 region, yet they will be ‘measured’ as a single product when using CD44v3 primers/probes/antibodies. Again, probes against any particular exons /protein domains od CD44 will demonstrate a wide range of CD44 isoforms, all of which share the same particular exon/domain. Furthermore, most of our tests are performed on mixtures of different tumour tissue elements: these assays work on a conglomerate of tumour cells, stromal cells, their proteins or gene products. Results of these reactions will lways represent some sort of average or summation. This might influence the qualitative expression pisture of distinct gene products of a tumour sample, but certainl will affect quantitative one. Considering this makes it easier to understand the contradictions of the literature.

Our aim was to identify a roster of CD44 isoforms expressed by colorectal cancer to see the extent of complexity we need to face when examining ‘CD44’. We also wanted to determine whether a potential “malignant” colorectal adenocarcinoma-specific isoform parttern exists. We then aimed to examine the stability of this pattern during tumour progression, i.e. whether it is qualitatively stable or new isoforms become dominant. This was tested in our complex iso- and xenograft experimental animal models. Reproducibility of v3 and v6 changes described in the literature was also tested in our experimental animal models. These models enabled us to separately study steps of tumour progression as well as the role of the tumour microenvironment.

First of all we attempted to identify the expressed isoforms from the qualitative CD44 ASP. We concluded that although not all the possible isoforms are expressed in reality, it is still rather technically impossible to identify the ones that do get expressed.All three attempts of ours on completing the isoform roster were only partially successful: (i) Our A – B primer overlapped the whole CD44 variable region, still just a few “dominant” isoforms could be identified as qualitative PCR products with direct sequencing. Some other isoforms could me identified according to the PCR product length, as well. Further CD44 variants were added by four other PCRs carried out with primer pairs designed onto particular exons of interest (v3 and v6). (ii) Next-generation sequencing theoretically should provide an allele-specific list of all transcribed isoforms. Still we reached limitations of the technique, but managed to identify more, sofar unidentified isoforms. (iii) I our previous publication on similar experimental set-up on melanoma (data under publication, Rásó-Barnett et al., paper accepted) identification attempt of “each” expressed CD44 isoform by cloning was reported to be fruitful, but again far from complete. We feel that the result of CD44 splicing is far richer than the sensitivity and effectivity of today’s tests. However, we do not yt know if this complexity of CD44 ASP carries any special functional importance. For now, we need to focus on functional characterization of distinct exons/domains of the complex molecule selectively.

Although the complete CD44 isoform list was not achieved, we identified simplified colorectal adenocarcinoma specific CD44 alternative splice pattern (ASP) which was qualitatively stable across samples from different colorectal cancer cell lines in vitro, and subcutaneous, intrasplenic and orthotopic implanted primary colorectal tumours and their liver metastases/colonies.

Although we know that not all of the expressed isoforms can be detected this way, the overlapping primer pairs we used covered the whole variable region, and provided a simplified pattern (ASP) which could be easily used to follow the possible changes of CD44 spilicing even in more complex in vivo systems. These results proved that, unlike the appearance of new CD44 isoforms during tumorigenesis, no new CD44 isoforms appear during tumour progression from primary tumour to metastasis. Therefore, it was likely that the expression changes of specific variant containing isoforms (as published in the literature) should be rather caused by *quantitative* changes of a set of isoforms.

Hence, we focused on quantitative expression of two particular exons: v3 and v6. These two exons also appeared to be dominant on our reported qualitative ASP pictures. We separately examined their role in different steps of metastatic cascade in our complex animal experimental model system. Furthermore we managed to gain a glimpse of the role of tumour microenvironment (host) in the modulation of CD44 splice variant expression in conjunction with metastatic potential, as well.

Our isograft model (Balb/C – C26) proved that quantitative changes only happen during real metastasis formation, considering that v3 and v6 expression levels of both orthotopic and heterotopic implanted primary tumours were similar, but increased expression levels were only detected in real liver metastases and not in the colonies. This means that the higher expression level of v3 and v6 is not the effect of the liver microenvironment, but it is the actual pre-existing feature of distinct metastatic cells within the primary tumour. While at orthotopic implantation into large bowel wall tumour cells must undergo each classical steps of metastasis formation (EMT, intravasation, survival in circulation, extravasation, MET, proliferation in metastatic target organ), intrasplenic implantation gives the chance to a proportion of tumour cells to get straingt into the circulation by skipping the early phases of the cascade. Comparison of the two models can potentially differenciate between genes, gene products that act in different in different phases of metastatic process.

The same theory was tested in a human xenograft model as well. HT29 human colorectal adenocarcinoma xenograft showed similar results to the C26 isograft, while both HT25 and HCT116 behaved differently, which proposed further explanation to the contradictions of CD44 literature. We hypothesise that a higher v3/v6 expression level is a necessary, but probably not satisfactory prerequisite of cellular metastatic capability. This is proven by the fact that real liver metastases of all three CRC cell lines expressed v3 and v6 in the same range, despite the different expression levels of their colonic primaries Figure 4A,B. This also means, that the number of cells expressing high levels of CD44v3 and v6 is not necessarily high within the primary tumour, as theoretically a single ‘highly expressing’ clone is enough for metastasis formation. In a similar experimental model system circulating tumour cells of malignant melanoma were found to express CD44v3 and v6 containing varints on a high level in real metastatic system (Rásó-Barnett et al. under publication) Correlating this with our result would mean that HT29 had the lowest, while HT25 and HCT116 had the higher percentage of ‘highly expressing’, metastatically potent cells. This also carries the possibility of a primary tumour showing relatively higher expression levels than its metastasis, which would have already been re-selected by the new microenvironment. Translating this into clinical practice would mean, that the ‘lack’ of overexpression would not exclude metastatic potential or even a worse prognosis. As primary tumour site seemed to be interesting in terms of selection of metastatically potent, CD44v3/v6 high subclones, we tried to find support os this theory in another, quite different model system.

We further examined the role of the primary tumour microenvironment in our subcutaneously implanted xenograft model. CD44v3 and v6 expression levels were different in tumours implanted from the same cell suspension, into genetically identical host, i.e. newborn and adult *scid* mice, which were only physiologically different. Hence, differences in expression levels of the examined gene products (v3 and v6) could be correlated with differences in metastatic potential (0% vs. 100%). Microenvironmental (host) factors (such as maturity immune system, vascular permeability, cytokines and chemokines, etc.) should, therefore, stand as driving factors in the background of metastatically potent subclones which will ultimately determine the clinical behaviour of the entire tumour. These experiments prove that physiological factors of host (as well as primary tumour microenvironment) do matter and in extreme, experimental conditions can fully determine metastatic potential of a malignant system.

## Conclusions

It is likely to assume that tumour microenvironment (tumour host organism) have a central role in metastatic phenotype presentation of primary colorectal cancer.

While normal tissues (such as colonic mucosa) do not express variant isoforms of CD44, tumours seem to perform a wide variation of CD44 isoforms. Presuming that different domains of different CD44 isoforms can perform a variety of new functions, there is no more sense in investigating CD44 “in general”.

Moreover, *expression pattern of CD44 isoforms was found to be stable*, meaning that colorectal type expression profile remained unchanged from tumour cell suspensions *(in vitro) throughout primary and metastatic colon cancer* xenografts. In contrary, quantitative changes do exist in the expression of distinct variant exons.

CD44 variant isoforms, especially the functionally well-characterized *v3 and v6* containing isoforms, seem to massively *take part in expressing the metastatic phenotype*. Our results support that higher-level v3/v6 co-expression can represent this “quasy-metastatic-gene” function at the primary tumour site and at the early phase of the metastatic cascade. Nontheless, it must be emphasized that only a minor proportion of the primary tumour mass is sufficient to hold “metastatic-phenotype” for the complete primary tumour. Although each colorectal tumour types use metastatic subclones of high CD44v3/v6 expression rate, selective examination of metastatic tumour cell group is not yet resolved. This means that quantitative evaluation of not only CD44 “in general”, but even of v3/v6-containing isoforms are inappropriate for the prognosis of metastatic behavior of a single tumour case because of the summative way of the measurement techniques. Moreover this can be the background of the quite diversive results on the predictive value of “CD44” in the literature.

## Methods

### Tumour cell cultures

We maintained cell cultures of three genetically different human colorectal cell lines (HT25 (from M.Hendricks, Iowa), HT29 (ECACC 91072201) and HCT116 (ICLC HTL95025)), four other human neoplastic cell lines (MCF7 – human breast cancer cell line (ATCC HTB-22^™^), K562 – chronic myelogenous leukemia cell line (ATCC CCL-243^™^), A431 – human vulvar epidermoid carcinoma cell line (ATCC CRL 1555), HT199 – human malignant melanoma cell line (developed in the 1st Department of Pathology and Experimental Cancer Research (Semmelweis University, Budapest, Hungary)) and a mouse colorectal cell line (C26 - derived from BALB/C Colon26 murine colon adenocarcinoma - obtained from ATCC. USA) in 5% FCS and 1% Penicillin/Streptomycin containing 1640-RPMI medium at 37°C. For implantation we prepared single-cell suspensions after enzymatic (Tripsine-EDTA) digestion and washed the cultures twice with FCS-free medium.

### In vivo experimental models

#### Isograft model

**Figure 5:**
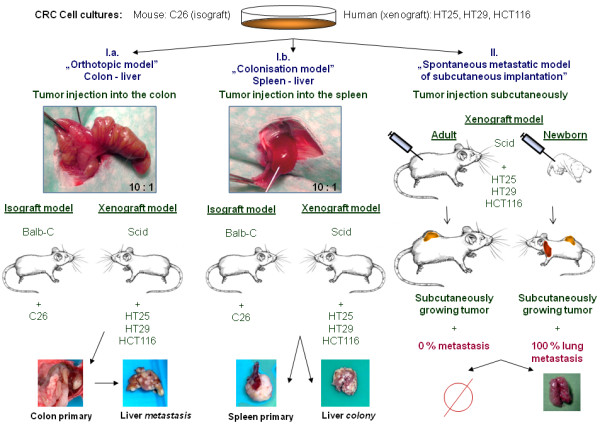
**Complex animal model system.** I. Liver metastasis (I.a) / colonisation (I.b) model. Human xenograft colon cancer cell suspensions (HT29, HCT116 and HT25) were intracolonic and intrasplenic injected to scid mice; C26 mouse isograft colon cancer cell suspension was injected into Balb/C mice. In proper „metastasis” system the whole metastatic sequence is represented, while „colonisation” model represents just the later part of metastatic cascade. II. Spontaneous lung metastatsis model of subcutaneous (heterotopic) tumour injection: single cell suspension of human xenograft colon cancer was injected into adult and newborn scid mice. Primary tumours were grown in both cases, while pulmonary metastases were produced only in the newborn.

We injected suspensions of C26 isograft colorectal tumour cell line into the spleen (4 animals) [80]and colonic wall (4 animals) of adult (20-week-old, 20g – weigh) Balb-C mice. Anaesthesia was performed by intraperitoneal injection of Nembuthal (70 mg/kg). Each tumour injection was performed by 0.05 ml suspension of 5x10^5^ cells (10^7^ cell/ml serum-free one-cell suspensions). After 4 weeks, animals were sacrificed, autopsy was performed and tumour tissues of primary tumours and liver metastases were isolated Figure 5I.a,I.b.

#### Human xenograft model I. - spontaneous lung metastatic model from heterotopic (subcutaneous) implantation

Single cell suspension of HT25, HT29 and HCT116 cell lines (5x10^5^ cells / 0.05 ml) were subcutaneously implanted into adult and newborn *scid* mice into the same localization (left lumbar region) using the same cell suspensions. Both primary and metastatic tumours were dissected from the sacrificed animals after 3 weeks Figure 5II. This model provides particular chance to examine “host” driven changes in the behavior of cancer cell suspensions: while in newbrn mice pulmonary metastases are being formed in each experimental cases, the same cell suspensions are never becoming “metastatic” after implantation into adult mice. The two systems (permissive and non-permissive) were only different in “host age”.

Micrometastases were verified on frozen sections of the lung.

#### Human xenograft model II - Liver-metastasis models from orthotopic and heterotopic implantation

We performed xenotransplantation of the already mentioned three human colorectal cancer cell lines (5x10^5^ cells / 0.05 ml) into the spleen (heterotopic implantation) and into the colonic wall (orthotopic implantation) of 20-25g-weigh adult *scid* mice. The operations were performed under intraperitoneal anaesthesia with 70 mg/kg Nembutal. Animals were sacrificed after 8–10 weeks when obvious signs of cachexia showed (more than 20% of weight loss of animal occurred). Primary and metastatic tumours were dissected during autopsy Figure 5I.a,I.b.

Samples from primary and metastatic tumours of all three experimental animal models were processed by freezing them immediately after removal for molecular biology.

All animal experiments have been approved by the local Animal Experimental Research Board (TUKEB83/2009).

### Total RNA isolation

Tumour tissue was fronzen in liqued nitrogen and homogenized in mortar prior to RNA isolation. Total RNA was isolated with Trizol® (SIGMA) according to manufacturer’s instructions. Possible DNA contamination was eliminated with TURBO DNA-free™ (Ambion).

### Reverse transcription

For reverse transcription 1 μl of 10mM dNTP mix (Finnzyme®) and 1μl of random nonamer-oligo dT combination for a final concentration of 2.5 μM was added to 2 μg of purified total RNA. After incubating at 70°C for 10 min 2 μl of 10x M-MLV Reverse Transcriptase Buffer (Finnzyme®), 1μl of M-MLV Reverse Transcriptase (200 units/μl, Finnzyme®), 0.5 μl RNase Inhibitor (40 units/μl, Promega®) and 6.5 μl RNAse free water was added for the final volume of 20 μl than incubated at 37°C for 50 min and 85°C for 10 min.

For checking the occurance of reverse transcription, β-actin was used as a housekeeping gene (βS1: 5’– TCT GGC ACC ACA CCT TCT AC–3’ and βA4: 5’–CTC CTT AAT GTC ACG CAC GAT TTC–3’, for murine tumours: MßS1(RefSeq NM_007393):5’-AGA CAA CAT TGG CAT GGC TT-3’, MßA1:5’-AAT GAA GTA TTA AGG CGG AAG ATT-3’), respectively). Under the same PCR conditions, RNA of the same sample was used as negative control for detecting DNA contamination and DEPC treated, RNAse free water as non-template control.

### PCR analysis

For investigation of the variable regions of CD44, serial PCR reactions were carried out with five different human-specific primer pairs (**A:**Standard 5’ : AGT CAC AGA CCT GCC CAA TGC CTT T, **B:**Standard 3’: TTT GCT CCA CCT TCT TGA CTC CCA TG, **C:**v3 exon 5’: GGG AGC CAA ATG AAG AAA ATG AAG ATG AAA G, **D:**v3 exon 3’ : GGT GCC TGT CTC TTT CAT CTT CAT TTT CTT CAT TT, **E:**v6 exon 3’: TCT GTT GCC AAA CCA CTG TTC CTT CTG) [22] Figure 2A.

The PCR reaction mixture contained 2.5 μl 10X PCR buffer+Mg2+ (DyNazymeTM), 2 μl dNTP mix (2.5 mM each), 0.4 μl DNA polymerase (DyNazymeTM, 2 U/μl), 2.5-2.5 μl of the primers, 2 μl of the cDNA and 13.1 μl DEPC treated water for the final volume of 25 μl. The cycling conditions were: 94°C for 12min once, then 94°C for 1 min, 65°C for 1 min, 72°C for 1 min for 40 cycles and the extension step was 72°C for 10 min.

PCR products were separated on a 3% agarose gel and detected with Gel Doc 2000 (Bio-Rad®) after ethidium bromide staining.

Transcribed isoforms were identified by re-extraction, and direct sequencing (Big Dye Terminator cycle sequencing, Applied Biosystems 3130 Genetic Analyzer) of the distinct bands.

### Quantitative PCR analysis

For quantitative measurement of CD44v3 and v6 variable exons q-PCR reactions were used. Each 25 μl reaction mixture contained 12.5 μl 2X iQ SYBR® Green Supermix (Bio-Rad), 0.5-0.5 μl of each primer for final concentration of 200nM and 11.5 μl of the diluted cDNA. The cycling conditions comprised 3 min of iTaq™ DNA polymerase activation at 95°C, 40 cycles at 95°C for 30 sec, 55°C for 30 sec, 72°C for 1 min. Starting quantities were defined on the basis of standard five-fold dilution series (x1-625) carried out with control cDNA of A431 (human squamous cell carcinoma). Relative expression of the examined v3 (primers: CGT CTT CAA ATA CCA TCT CAG CA and ATC TTC ATC ATC AAT GCC TGA) and v6 (primers: GGC AAC TCC TAG TAG TAC AAC G and GTC TTC TCT GGG TGT TTG GC) variable exons were determined by normalizing the starting quantities to the housekeeping β-actin (primers: GTG GGG CGC CCC AGG CAC CA and CTC CTT AAT GTC ACG CAC GAT TTC) starting quantities from the same cDNA sample.

### Next-generation, allele-specific sequencing

In order to prove identified CD44 isoforms specific to colorectal cancer, next-generation sequencing method was performed. The method briefly is as follows: PCR reaction products from HT29, HT25 and HCT116 human colorectal cell lines (total RNA isolation, purification, reverse transcription as mentioned above) with primer pair A – B (primers designed on 5’ and 3’ standard region of CD44 framing the entire variable region, hence theoretically amplifying all the transcribed variant isoforms at the same time) were first amplified, purified with High pure PCR clean-up micro kit (Roche®, Mannheim) according to the manufacturer’s instructions. The amplicon ends were polished and ligated with roche 454 multiplex identifier (MID) adaptors, so that universal primer binding sites were generated for emulsion PCR. The amplicons were than separated with magnetic ampure beads (Agencourt), non-bound adaptors were eliminated. Emulsion PCR (emPCR) reaction was performed with molarly equivalent amount of sample amplicons according to the manufacturer’s protocol (Roche 454®) in modified condition of low A,B primer concentration and slow PCR program to minimalize the amplification efficiency differences between longer and shorter variants. CD44 variant isoform library was clonally amplified with pyrosequencing technique (454 GS Junior, Roche®), under the condition of 5% bead-ratio, 200 sequencing cycles, 400 bases read length mode.

Amplicon variant analyzer software (Roche Diagnostics®) was used to eliminate multiplicity of the samples after MIDs, to trim the primers and to align the reads to the reference cDNA sequence. For easier handling of the huge database of sequenced isoforms of the critically long CD44 molecule, exon-by-exon fishing technique was adapted to identify each amplified exon-combination splice variants.

Aligment of an exon was accepted to be valid over a threshold of matching rate of 90%. The exon combinations which have more than 50 reads were reported.

Similar process was performed by the four other primer combinations (Primer A-D, A-E, C-B and C-E, respectively. See Figure 2A and Methods above).

### Statistical analysis

Real-time PCR results, relative expression rates (normalized on beta-actin expression rates) were compared with two-sample T-tests (data were showing normal distribution). Statistical significance was assumed at p< 0.05.

## Abbreviations

ASP: Alternative splice pattern; bp: Base pair; CRC: Colorectal cancer; DEPC: Diethylpyrocarbonate; EDTA: Ethylenediaminetetraacetic acid; ECM: Extracellular matrix; ELFO: Electrophoresis; emPCR: Emulsion polymerase chainreaction; EMT: Epithelial-mesnchymal transition; FCS: Foetal calf serum; MET: Mesenchymal-epithelial transition; NGS: Next-generation sequencing; PCR: Polymerase chainreaction; q-PCR: Quantitative polymerase chainreaction; scid: Severe combined immunodeficiency (mouse).

## Competing interests

There is no competing interest affecting the authors.

## Authors’ contributions

BB and LRB designed and described CD44 ASP, complex experimental animal model system was deisgned by ER, isograft animal experiments, as well as spontaneous lung metastasis experimets were carried out by BT and RE, while orthotopic and colonization xenograft models were carried out by BB. TB and LRB participated in designing and performing qPCR studies, too. PB was performing next-generation sequencing on the basis of the experimental design of ER. Statistical assessment, interpretation of result data were led by ER and JT and performed by BB. Direct sequencing was carried out by TB and ER. ER and JT helped in drafting the manuscript. All authors read and approved the final manuscript.

## Grant Support

This study was supported by grants from the TÁMOP-4.2.1./B-09/1/KMR-2010-0001 and ETT 087-07/2009.

## References

[B1] Parkin DM Bray F Ferlay J Pisani P Global cancer statistics, 2002 CA Cancer J Clin 2012 55 74 108 15761078 10.3322/canjclin.55.2.74

[B2] Gryfe R Swallow C Bapat B Redston M Gallinger S Couture J Molecular biology of colorectal cancer Curr Probl Cancer 2012 21 233 300 9438104 10.1016/s0147-0272(97)80003-7

[B3] Kim H Yang XL Rosada C Hamilton SR August JT CD44 expression in colorectal adenomas is an early event occurring prior to K-ras and p53 gene mutation Arch Biochem Biophys 1994 310 504 507 10.1006/abbi.1994.1199 7513984

[B4] Hao H Muniz-Medina VM Mehta H Thomas NE Khazak V Der CJ Shields JM Context-dependent roles of mutant B-Raf signaling in melanoma and colorectal carcinoma cell growth Mol Cancer Ther 2007 6 2220 2229 10.1158/1535-7163.MCT-06-0728 17699719

[B5] Fearon ER Vogelstein B A genetic model for colorectal tumorigenesis Cell 1990 61 759 767 10.1016/0092-8674(90)90186-I 2188735

[B6] Djebali S Davis CA Merkel A Dobin A Lassmann T Mortazavi A Tanzer A Lagarde J Lin W Schlesinger F Xue C Marinov GK Khatun J Williams BA Zaleski C Rozowsky J Röder M Kokocinski F Abdelhamid RF Alioto T Antoshechkin I Baer MT Bar NS Batut P Bell K Bell I Chakrabortty S Chen X Chrast J Curado J Landscape of transcription in human cells Nature 2012 489 101 108 10.1038/nature11233 22955620 PMC3684276

[B7] Lyons AJ Jones J Cell adhesion molecules, the extracellular matrix and oral squamous carcinoma Int J Oral Maxillofac Surg 2007 36 671 679 10.1016/j.ijom.2007.04.002 17643963

[B8] Kanke M Fujii M Kameyama K Kanzaki J Tokumaru Y Imanishi Y Tomita T Matsumura Y Role of CD44 variant exon 6 in invasion of head and neck squamous cell carcinoma Arch Otolaryngol Head Neck Surg 2000 126 1217 1223 11031408 10.1001/archotol.126.10.1217

[B9] Rodrigues LR Teixeira JA Schmitt FL Paulsson M Lindmark-Mänsson H The role of osteopontin in tumor progression and metastasis in breast cancer Cancer Epidemiol Biomarkers Prev 2007 16 1087 1097 10.1158/1055-9965.EPI-06-1008 17548669

[B10] Faleiro-Rodrigues C Lopes C E-cadherin, CD44 and CD44v6 in squamous intraepithelial lesions and invasive carcinomas of the uterine cervix: an immunohistochemical study Pathobiology 2004 71 329 336 10.1159/000081729 15627844

[B11] Vihinen PP Pyrhönen SO Kähäri V-M New prognostic factors and developing therapy of cutaneous melanoma Ann Med 2003 35 66 78 10.1080/07853890310009980 12795336

[B12] Ermak G Jennings T Robinson L Ross JS Figge J Restricted patterns of CD44 variant exon expression in human papillary thyroid carcinoma restricted patterns of CD44 variant exon expression in human papillary thyroid Cancer Res 1996 56 1037 1042 8640758

[B13] Tahara E Molecular biology of gastric cancer World J Surg 1995 19 484 488 10.1007/BF00294705 7676688

[B14] Wittig BM Goebel R Weg-Remers S Pistorius G Feifel G Zeitz M Stallmach A Stage-specific alternative cplicing of CD44 and alpha 6 beta 1 integrin in colorectal tumorigenesis Exp Mol Pathol 2001 70 96 102 10.1006/exmp.2000.2337 11263953

[B15] Wielenga VJ van der Voort R Taher TE Smit L Beuling EA van Krimpen C Spaargaren M Pals ST Expression of c-Met and heparan-sulfate proteoglycan forms of CD44 in colorectal cancer Am J Pathol 2000 157 1563 1573 10.1016/S0002-9440(10)64793-1 11073815 PMC1885727

[B16] Yamada Y Itano N Narimatsu H Kudo T Hirohashi S Ochiai A Niimi A Ueda M Kimata K Receptor for hyaluronan-mediated motility and CD44 expressions in colon cancer assessed by quantitative analysis using real-time reverse transcriptase-polymerase chain reaction Jpn j cancer res 1999 90 987 992 10.1111/j.1349-7006.1999.tb00846.x 10551329 PMC5926169

[B17] Aruffo A Stamenkovic I Melnick M Underhill CB Seed B CD44 is the principal cell surface receptor for Cell 1990 61 1303 1313 10.1016/0092-8674(90)90694-A 1694723

[B18] van Royen N Voskuil M Hoefer I Jost M de Graaf S Hedwig F Andert J-P Wormhoudt TaM Hua J Hartmann S Bode C Buschmann I Schaper W van der Neut R Piek JJ Pals ST CD44 regulates arteriogenesis in mice and is differentially expressed in patients with poor and good collateralization Circulation 2004 109 1647 1652 10.1161/01.CIR.0000124066.35200.18 15023889

[B19] Brennan FR Mikecz K Glant TT Jobanputra P Pinder S Bavington C Morrison P Nuki G CD44 expression by leucocytes in rheumatoid arthritis and modulation by specific antibody: implications for lymphocyte adhesion to endothelial cells and synoviocytes in vitro Scand J Immunol 1997 45 213 220 10.1046/j.1365-3083.1997.d01-382.x 9042434

[B20] Kitano A Oshitani N Matsumoto T Kobayashi K Display Settings: CD44 variants in ulcerative colitis and Crohn ’ s disease Lancet 1996 348 266 267 8684214 10.1016/s0140-6736(05)65573-0

[B21] Teder P Vandivier RW Jiang D Liang J Cohn L Puré E Henson PM Noble PW Resolution of lung inflammation by CD44 Science 2002 296 155 158 10.1126/science.1069659 11935029

[B22] Givehchian M Woerner SM Lacroix J Zöller M Drings P Becker H Kayser K Ridder R von Knebel Doeberitz M Expression of CD44 splice variants in normal respiratory epithelium and bronchial carcinomas: no evidence for altered CD44 splicing in metastasis Oncogene 1996 12 1137 1144 8649806

[B23] Grimme HU Termeer CC Bennett KL Weiss JM Schöpf E Aruffo A Simon JC Colocalization of basic fibroblast growth factor and CD44 isoforms containing the variably spliced exon v3 (CD44v3) in normal skin and in epidermal skin cancers Br J Dermatol 1999 141 824 832 10.1046/j.1365-2133.1999.03154.x 10583162

[B24] Isabel Fonseca JFMN JS Expression of CD44 isoforms in normal salivary gland tissue: an immunohistochemical and ultrastructural study Histochem Cell Biol 2000 114 5 6 10.1007/s004180000220 11201610

[B25] Fox SB Fawcett J Jackson DG Collins I Gatter KC Harris AL Gearing A Simmons DL Normal human tissues, in addition to some tumors, express multiple different CD44 isoforms normal human tissues, in addition to some tumors, express multiple Cancer Res 1994 54 4539 4546 7519124

[B26] Woerner SM Givehchian M Dürst M Schneider A Costa S Melsheimer P Lacroix J Zöller M Doeberitz MK Expression of CD44 splice variants in normal, dysplastic, and neoplastic cervical epithelium Clin Cancer Res 1995 1 1125 1132 9815903

[B27] Levin TG Powell AE Davies PS Silk AD Dismuke AD Anderson EC Swain JR Wong MH Characterization of the intestinal cancer stem cell marker CD166 in the human and mouse gastrointestinal tract Gastroenterology 2010 139 2072 2082.e5 10.1053/j.gastro.2010.08.053 20826154 PMC2997177

[B28] Zeilstra J Joosten SPJ Dokter M Verwiel E Spaargaren M Pals ST Deletion of the WNT target and cancer stem cell marker CD44 in Apc(Min/+) mice attenuates intestinal tumorigenesis Cancer Res 2008 68 3655 3661 10.1158/0008-5472.CAN-07-2940 18483247

[B29] Vaiopoulos AG Kostakis ID Koutsilieris M Papavassiliou AG Colorectal cancer stem cells Stem cells 2012 30 363 371 10.1002/stem.1031 22232074

[B30] Asao T Nakamura J Shitara Y Tsutsumi S Mochiki E Shimura T Takenoshita S Kuwano H Loss of standard type of CD44 expression in invaded area as a good indicator of lymph-node metastasis in colorectal carcinoma Dis Colon Rectum 2000 43 1250 1254 discussion 1254–5 10.1007/BF02237430 11005492

[B31] Wielenga VJM Heider K Johan G Progression T Offerhaus GJA Adolf G Berg FMVD Punta H Herrlich P Pals ST Expression of CD44 variant proteins in human colorectal cancer is related to tumor progression advances in brief expression of CD44 variant proteins in human colorectal cancer is related to Cancer Res 1993 53 4754 4756 7691404

[B32] Zhu J He J Liu Y Simeone DM Lubman DM Identification of glycoprotein markers for pancreatic cancer CD24+CD44+ stem-like cells using nano-LC-MS/MS and tissue microarray J Proteome Res 2012 11 2272 2281 10.1021/pr201059g 22335271 PMC3321127

[B33] Dalerba P Dylla SJ Park I-K Liu R Wang X Cho RW Hoey T Gurney A Huang EH Simeone DM Shelton AA Parmiani G Castelli C Clarke MF Phenotypic characterization of human colorectal cancer stem cells Proc Natl Acad Sci U S A 2007 104 10158 10163 10.1073/pnas.0703478104 17548814 PMC1891215

[B34] Alderton G Tumour stem cells: generating colon cancer Nat Rev Cancer 2006 6 906 907

[B35] Fillmore C Kuperwasser C Human breast cancer stem cell markers CD44 and CD24: enriching for cells with functional properties in mice or in man? Breast Cancer Res 2007 9 303 10.1186/bcr1673 17540049 PMC1929090

[B36] Takaishi S Okumura T Tu S Wang SSW Shibata W Vigneshwaran R Shanisha AK Gordon SA Shimada Y Wang TC Identification of Gastric Cancer Stem Cells Using the Cell Surface Marker CD44 Stem Cells 2009 27 1006 1020 10.1002/stem.30 19415765 PMC2746367

[B37] Li C Heidt DG Dalerba P Burant CF Zhang L Adsay V Wicha M Clarke MF Simeone DM Identification of pancreatic cancer stem cells Cancer Res 2007 67 1030 1037 10.1158/0008-5472.CAN-06-2030 17283135

[B38] Koukourakis MI Giatromanolaki A Tsakmaki V Danielidis V Sivridis E Cancer stem cell phenotype relates to radio-chemotherapy outcome in locally advanced squamous cell head-neck cancer Br J Cancer 2012 106 846 853 10.1038/bjc.2012.33 22333601 PMC3305970

[B39] Hu Y Fu L Targeting cancer stem cells: a new therapy to cure cancer patients Am J Cancer Res 2012 2 340 356 22679565 PMC3365812

[B40] Tumor M Lilly P Bourguignon W CD44 in Cancer Progression: adhesion Migration and Growth Regul 2004 35 211 231 10.1023/b:hijo.0000032354.94213.69 15339042

[B41] Kuhn S Koch M Nübel T Ladwein M Antolovic D Klingbeil P Hildebrand D Moldenhauer G Langbein L Franke WW Weitz J Zöller M A complex of EpCAM, claudin-7, CD44 variant isoforms, and tetraspanins promotes colorectal cancer progression Mol Cancer Res 2007 5 553 567 10.1158/1541-7786.MCR-06-0384 17579117

[B42] Ngan CY Yamamoto H Seshimo I Ezumi K Terayama M Hemmi H Takemasa I Ikeda M Sekimoto M Monden M A multivariate analysis of adhesion molecules expression in assessment of colorectal cancer J Surg Oncol 2007 95 652 662 10.1002/jso.20638 17443723

[B43] Camp RL Kraus TA Puré E Variations in the cytoskeletal interaction and posttranslational modification of the CD44 homing receptor in macrophages J Cell Biol 1991 115 1283 1292 10.1083/jcb.115.5.1283 1955476 PMC2289237

[B44] Bendardaf R Algars A Elzagheid A Korkeila E Ristamäki R Lamlum H Collan Y Syrjänen K Pyrhönen S Comparison of CD44 expression in primary tumours and metastases of colorectal cancer Oncol Rep 2006 16 741 746 16969488

[B45] Marhaba R Zöller M CD44 in cancer progression: adhesion, migration and growth regulation J Mol Histol 2004 35 211 231 15339042 10.1023/b:hijo.0000032354.94213.69

[B46] Fujisaki T Tanaka Y Fujii K CD44 stimulation induces integrin-mediated adhesion of colon cancer cell lines to endothelial cells by up-regulation of integrins and c-met and activation of integrins CD44 stimulation induces integrin-mediated adhesion of colon cancer cell lines Cancer Res 1999 59 4427 4434 10485493

[B47] Ghatak S Misra S Toole BP Hyaluronan oligosaccharides inhibit anchorage-independent growth of tumor cells by suppressing the phosphoinositide 3-kinase/Akt cell survival pathway J Biol Chem 2002 277 38013 38020 10.1074/jbc.M202404200 12145277

[B48] Kuniyasu H Oue N Tsutsumi M Tahara E Yasui W Heparan sulfate enhances invasion by human colon carcinoma cell lines through expression of CD44 variant exon 3 Clin Cancer Res 2001 7 4067 4072 11751503

[B49] Ohashi R Takahashi F Cui R Yoshioka M Gu T Sasaki S Tominaga S Nishio K Tanabe KK Takahashi K Interaction between CD44 and hyaluronate induces chemoresistance in non-small cell lung cancer cell Cancer Lett 2007 252 225 234 10.1016/j.canlet.2006.12.025 17276588

[B50] Klingbeil P Marhaba R Jung T Kirmse R Ludwig T Zöller M CD44 variant isoforms promote metastasis formation by a tumor cell-matrix cross-talk that supports adhesion and apoptosis resistance Mol Cancer Res 2009 7 168 179 10.1158/1541-7786.MCR-08-0207 19208744

[B51] Goodison S Urquidi V Tarin D CD44 cell adhesion molecules Mol Pathol 1999 52 189 196 10.1136/mp.52.4.189 10694938 PMC395698

[B52] Sneath RJ Mangham DC The normal structure and function of CD44 and its role in neoplasia Mol Pathol 1998 51 191 200 10.1136/mp.51.4.191 9893744 PMC395635

[B53] Naor D Nedvetzki S Golan I Melnik LFY Display Settings: CD44 in cancer Crit Rev Clin Lab Sci 2002 39 527 579 10.1080/10408360290795574 12484499

[B54] Naor D Sionov RV Ish-Shalom D CD44: structure, function, and association with the malignant process Adv Cancer Res 1997 71 241 319 9111868 10.1016/s0065-230x(08)60101-3

[B55] Skotheim RI Nees M Alternative splicing in cancer: noise, functional, or systematic? Int J Biochem Cell Biol 2007 39 1432 1449 10.1016/j.biocel.2007.02.016 17416541

[B56] Zalewski B Levels of v5 and v6 CD44 splice variants in serum of patients with colorectal cancer are not correlated with pT stage, histopathological grade of malignancy and clinical features World J Gastroenterol 2004 10 583 585 14966921 10.3748/wjg.v10.i4.583 PMC4716984

[B57] Zalewski B Stasiak-Barmuta A Sulkowski S Piotrowski Z Myśliwiec P Kukliński A Glycoprotein CD44 variant 4 expression in tumour epithelial cells of patients with colorectal cancer Rocz Akad Med Bialymst 2004 49 Suppl 1 37 39 15638368

[B58] Weg-Remers S Anders M von Lampe B Riecken EO Schüder G Feifel G Zeitz M Stallmach A Decreased expression of CD44 splicing variants in advanced colorectal carcinomas Eur J Cancer 1998 34 1607 1611 10.1016/S0959-8049(98)00177-4 9893637

[B59] Gutt CN Kim Z-G Schemmer P Krähenbühl L Schmedt C-G Impact of laparoscopic and conventional surgery on Kupffer cells, tumor-associated CD44 expression, and intrahepatic tumor spread Arch Surg 2002 137 1408 1412 10.1001/archsurg.137.12.1408 12470109

[B60] Bennett KL Jackson DG Simon JC Tanczos E Peach R Modrell B Stamenkovic I Plowman G Aruffo A CD44 isoforms containing exon V3 are responsible for the presentation of heparin-binding growth factor J Cell Biol 1995 128 687 698 10.1083/jcb.128.4.687 7532176 PMC2199889

[B61] Spix Julie K Chay Edward Y Block Ethan RKJK Hepatocyte growth factor induces epithelial cell motility through transactivation of the epidermal growth factor receptor Exp Cell Res 2007 313 3319 3325 10.1016/j.yexcr.2007.06.006 17643426 PMC2128736

[B62] Orian-rousseau V Chen L Sleeman JP Herrlich P Ponta H CD44 is required for two consecutive steps in HGF / c-Met signaling Genes Dev 2002 16 3074 3086 10.1101/gad.242602 12464636 PMC187488

[B63] Cheng C Yaffe MB Sharp PA A positive feedback loop couples Ras activation and CD44 alternative splicing Genes Dev 2006 20 1715 1720 10.1101/gad.1430906 16818603 PMC1522067

[B64] Hofmann M Rudy W Günthert U Giinthert U Zimmer SG Zawadzki V Zã M Lichtner RB Herrlich P Ponta H A link between ras and metastatic mehavior of tumor cells: ras induces CD44 promoter activity and leads to low-level expression of metastasis-specific variants of CD44 in CREF cells Cancer Res 1993 53 1516 1521 8453616

[B65] Reeder JA Gotley DC Walsh MD Fawcett J Antalis TM Expression of antisense CD44 variant 6 inhibits colorectal tumor metastasis and tumor growth in a wound environment expression of antisense CD44 variant 6 inhibits colorectal tumor metastasis and tumor growth in a wound environment1 Cancer Res 1998 58 3719 3726 9721884

[B66] Haruyama K Matsumura Y Moriya Y Kakizoe T Ochiai A Kawaguchi MST Clinicopathological significance of the expression of CD44v2 in colorectal cancer Anticancer Res 1999 19 4421 4428 10650786

[B67] Barbour AP Reeder JA Walsh MD Fawcett J Antalis TM Gotley DC Expression of the CD44v2-10 isoform confers a metastatic phenotype: importance of the heparan sulfate attachment site CD44v3 Cancer Res 2003 63 887 892 12591743

[B68] Ropponen KM Eskelinen MJ Lipponen PK Alhava E Kosma VM Expression of CD44 and variant proteins in human colorectal cancer and its relevance for prognosis Scand J Gastroenterol 1998 33 301 309 10.1080/00365529850170900 9548625

[B69] Yamada Y Itano N Narimatsu H Kudo T Hirohashi S Ochiai A Tohnai I Ueda M Kimata K CD44 variant exon 6 expressions in colon cancer assessed by quantitative analysis using real time reverse transcriptase-polymerase chain reaction Oncol Rep 2003 10 1919 1924 14534719

[B70] Dong W-G Sun X-M Yu B-P Luo H-S Yu J-P Role of VEGF and CD44v6 in differentiating benign from malignant ascites World J Gastroenterol 2003 9 2596 2600 14606105 10.3748/wjg.v9.i11.2596 PMC4656549

[B71] Morrin MDP CD44v6 is not relevant in colorectal tumour Int J Colorectal Dis 2002 17 30 36 10.1007/s003840100335 12018451

[B72] van Weering DH Baas PD Bos JL A PCR-based method for the analysis of human CD44 splice products PCR Methods Appl 1993 3 100 106 10.1101/gr.3.2.100 7505677

[B73] Heider K Dämmrich J Skroch-angel P Herrlich P Ponta H Differential expression of CD44 splice variants in intestinal- and diffuse-type human gastric carcinomas and normal gastric mucosa differential expression of CD44 splice variants in intestinal- and diffuse-type human gastric carcinomas and normal gastric Cancer Res 1993 53 4197 4203 7689929

[B74] Muthukumaran N Miletti-González KE Ravindranath AK Rodríguez-Rodríguez L Tumor necrosis factor-alpha differentially modulates CD44 expression in ovarian cancer cells Mol Cancer Res 2006 4 511 520 10.1158/1541-7786.MCR-05-0232 16908592

[B75] Shin C Manley JL Cell signalling and the control of pre-mRNA splicing Nat Rev Mol Cell Biol 2004 5 727 738 10.1038/nrm1467 15340380

[B76] Gotley DC Fawcett J Walsh MD Reeder JA Simmons DL Antalis TM Alternatively spliced variants of the cell adhesion molecule CD44 and tumour progression in colorectal cancer Br J Cancer 1996 74 342 351 10.1038/bjc.1996.364 8695347 PMC2074640

[B77] Khoursheed M Mathew TC Makar RR Sonia L Abul H Asfar S Al-Sayer H Dashti HM Al-Bader A Expression of CD44s in human colorectal cancer Pathol Oncol Res 2002 8 170 174 10.1007/BF03032390 12515996

[B78] Rudzki Z LeDuy L Jothy S Changes in CD44 expression during carcinogenesis of the mouse colon Exp Mol Pathol 1997 64 114 125 10.1006/exmp.1997.2214 9316589

[B79] Furuta K Zahurak M Yang XL Rosada C Goodman SN August JT Hamilton SR Relationship between CD44 expression and cell proliferation in epithelium and stroma of colorectal neoplasms Am J Pathol 1996 149 1147 1155 8863664 PMC1865185

[B80] Paku S Lapis K Morphological aspects of angiogenesis in experimental liver metastases Am J Pathol 1993 143 926 936 7689793 PMC1887218

